# Supplementary material for: Seminoma and Embryonal Carcinoma Footprints Identified by Analysis of Integrated Genome-Wide Epigenetic and Expression Profiles of Germ Cell Cancer Cell Lines
Source: PLoS One. 2014 Jun 2;9(6):e98330. doi: 10.1371/journal.pone.0098330 (PMC4041891; doi:10.1371/journal.pone.0098330)
Supplement: File S1 — ZIP file containing DMRforPairs output for significant regions. Please start from the html files. (ZIP) [file pone.0098330.s008.zip › figures/890.pdf]

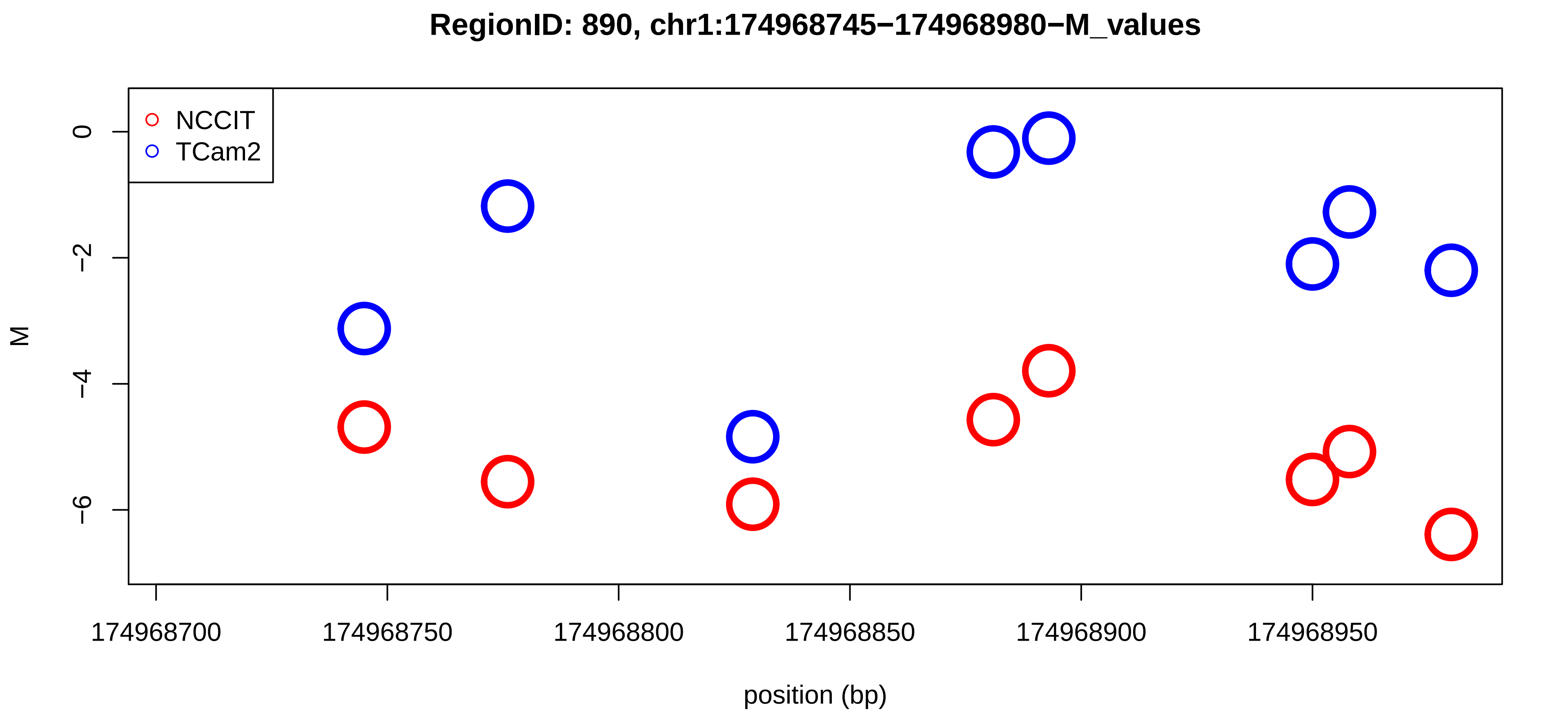

RegionID: 890, chr1:174968745−174968980−M_values
NCCIT
TCam2
0
−2
−4
−6
M
174968700
174968750
174968800
174968850
174968900
174968950
position (bp)

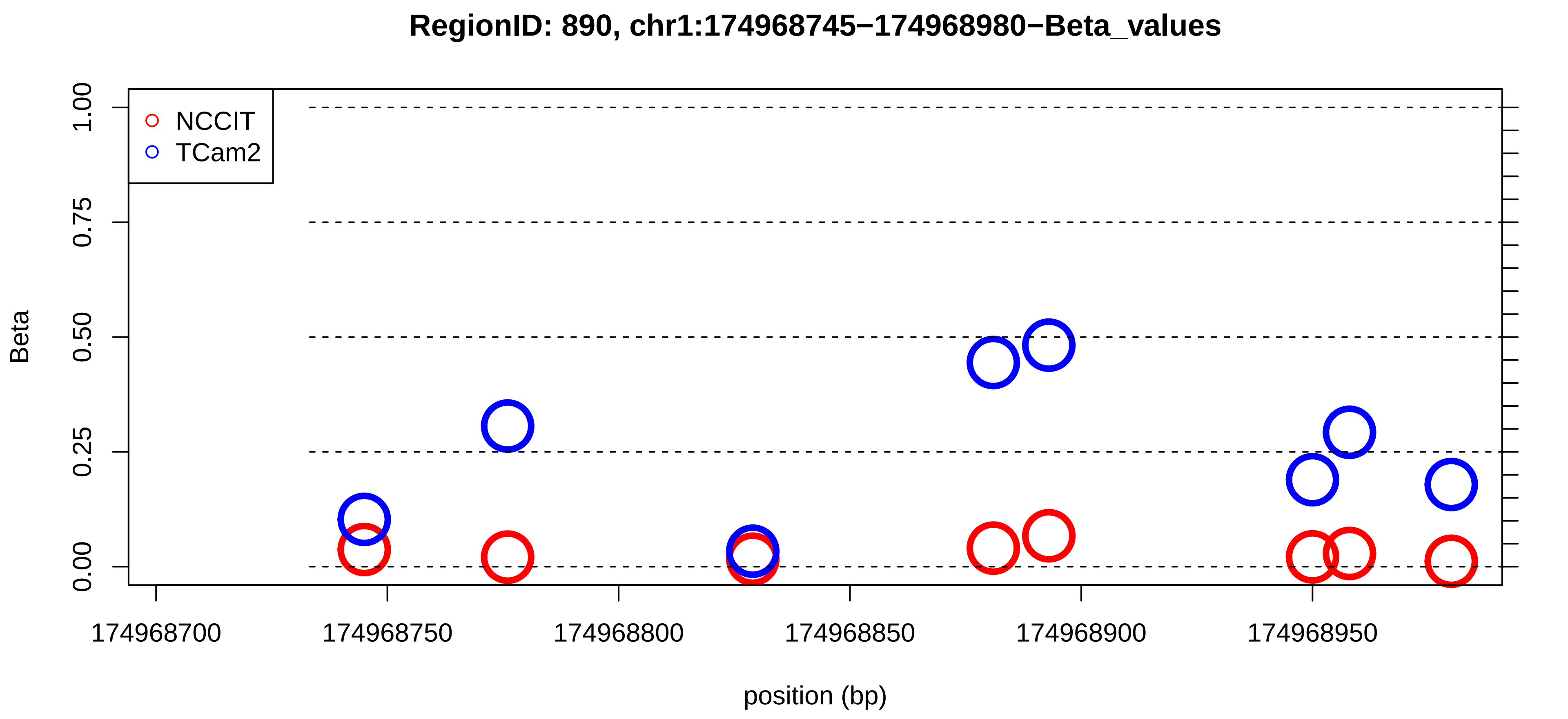

RegionID: 890, chr1:174968745−174968980−Beta_values
NCCIT
TCam2
1.00
0.75
0.50
0.25
0.00
Beta
174968700
174968750
174968800
174968850
174968900
174968950
position (bp)
